# Cerebrovascular remodeling in aging and neurodegenerative disease progression

**DOI:** 10.3389/fbioe.2025.1597917

**Published:** 2025-09-03

**Authors:** Samuel C. Halvorsen, Anastasia Gkousioudi, Raymond Nicks, Victor E. Alvarez, Irving J. Bigio, Joseph Zaia, Thor D. Stein, Yanhang Zhang

**Affiliations:** ^1^ Mechanical Engineering, Boston University, Boston, MA, United States; ^2^ Alzheimer’s Disease and CTE Research Center, Boston University Chobanian and Avedisian School of Medicine, Boston, MA, United States; ^3^ Biomedical Engineering, Boston University, Boston, MA, United States; ^4^ Biochemistry and Cell Biology, Boston University Chobanian and Avedisian School of Medicine, Boston, MA, United States; ^5^ United States Department of Veterans Affairs, VA Boston Healthcare System, Boston, MA, United States; ^6^ United States Department of Veterans Affairs, VA Bedford Healthcare System, Bedford, MA, United States; ^7^ Pathology and Laboratory Medicine, Boston University Chobanian and Avedisian School of Medicine, Boston, MA, United States; ^8^ Division of Materials Science and Engineering, Boston University, Boston, MA, United States

**Keywords:** anterior cerebral artery (ACA), Alzheimer’s disease, chronic traumatic encephalopathy (CTE), neuropathology, mechanical characterization, multiphoton imaging, aging

## Abstract

The cerebrovasculature is responsible for supplying oxygenated blood and nutrients to the brain and removing neurotoxic buildup. With age, trauma, and disease, the structural constituents of cerebral arteries including the extracellular matrix and smooth muscle cells are subject to remodeling and degradation. Cerebrovascular dysfunction can have detrimental impacts on the brain and is closely associated with cognitive impairment. Clinical studies have found that cerebrovascular dysfunction is correlated with cognitive decline in neurodegenerative diseases including Alzheimer’s disease (AD) and chronic traumatic encephalopathy (CTE). However, cerebrovascular changes during the progression of neurological disorders remain to be understood. Using matched and parallel studies of cerebrovasculature and brain tissue, this study set out to determine the temporal development of cerebrovascular remodeling and neurodegenerative disease progression. We examined changes to human anterior cerebral arteries (ACAs) from subjects with various degrees of AD and CTE neuropathology. Using biaxial inflation-extension testing, histological staining, and multiphoton imaging, we examined changes to the mechanical response and to the ACA wall structure. We found circumferential stiffening of the ACA with age. Furthermore, a minor relationship was reported between ACA stiffening and elevated levels of tau-based neuropathologies including neurofibrillary tangles, characteristic of both AD and CTE. Histological and multiphoton structural studies of the ACAs revealed smooth muscle cell atrophy at the media-adventitia interface and disorganization and straightening of adventitial collagen with age and disease. Our study reveals changes to the extracellular and cellular components of cerebral arteries that help describe the functional alterations of cerebrovasculature. Results from this study shed light on the complex relationship between cerebrovascular remodeling and neurodegenerative disease progression.

## Introduction

The cerebrovascular system plays a vital role in the body, distributing approximately 15%–20% of the cardiac output (Xing et al., 2017) through a network of vessels that extends 400 miles (Zlokovic, 2005) to supply the brain with oxygen, glucose, and other nutrients. Proper function of cerebral arteries is necessary to ensure ample brain tissue perfusion. With age and disease, cerebral vessels are subject to structural changes impacting biomechanical functions with an increased risk of cerebrovascular ischemia and infarctions (Lassen, 1959).

Cerebrovasculature function is closely associated with brain metabolism. Compliance of large cerebral arteries is critical to dampen pulsatile blood pressure, protecting the surrounding brain tissue and smaller vessels downstream (Kisler et al., 2017). Decreased cerebral artery compliance imparts unwanted physical forces onto the blood-brain barrier (BBB) within smaller vessels downstream (Kisler et al., 2017). Damage to the BBB compromises its ability to remove neurotoxic proteins, allowing them to accumulate in brain tissue and contribute to neurodegeneration and cognitive decline (Gorelick et al., 2011). Noninvasive clinical studies using magnetic resonance imaging and positron emission tomography have reported impaired cerebrovascular function as a risk factor for dementias including Alzheimer’s Disease (AD) (Stoquart-ElSankari et al., 2007; Uh et al., 2010; Roher et al., 2012). Despite the crucial role of the cerebrovasculature in brain function, little is known about changes to the microstructure and mechanical properties of human cerebral arteries resulting from age and neuropathological disease progression.

Affecting nearly seven million people in the United States, AD is the most common form of dementia and is characterized by gradual atrophy of brain tissue and cognitive decline (Alzheimer’s Association, 2024). Post-mortem diagnosis of AD progression is performed via histopathological examination of the spread of amyloid-β (Aβ) plaques and hyperphosphorylated tau (p-tau) in the form of neurofibrillary tangles (NFTs) throughout the brain (Hyman and Trojanowski, 1997; Hyman et al., 2012). Over time, these proteins accumulate throughout the brain and impair cognitive ability. Historically, AD was solely considered a consequence of these protein deposits, but recent studies suggest a direct association between cerebrovascular dysfunction and neuropathological AD progression (Kivipelto et al., 2001; Tong et al., 2005; Korczyn et al., 2012). Our recent study showed an association between AD progression and increased stiffness of human anterior cerebral arteries (ACAs) (Liu et al., 2023). Chronic traumatic encephalopathy (CTE) is another neurodegenerative disease defined by the accumulation of p-tau aggregates around blood vessels and at the sulcal depths as a result of repetitive head impacts (RHI) (McKee et al., 2013). Neurofibrillary tangles (NFTs) and other forms of p-tau are quantified post-mortem to stage CTE and are pathologically distinct from those in AD development (Stein et al., 2014; McKee et al., 2015). Although not required for diagnosis, Aβ deposition frequently occurs in CTE (McKee et al., 2015). Deposition of Aβ can also occur within small cerebral vessels, referred to as cerebral amyloid angiopathy (CAA) (Standring et al., 2019). CAA is nearly ubiquitous in AD and can present in CTE with an altered distribution (Standring et al., 2019).

Using matched and parallel groups of cerebrovasculature samples and brain tissues, we examined changes to the structure and function of the human anterior cerebral artery and their associations with aging and the progression of AD and CTE neuropathologies. Biaxial inflation-extension tests were performed to characterize the axial and circumferential responses during pressurization. To understand the relationship between cerebrovascular remodeling and neuropathological disease progression, the biomechanical testing results were first grouped by age and then by whole-brain neuropathologic measures associated with AD and CTE to establish associations between ACA wall mechanics and brain pathology. Histological staining and multiphoton imaging were then performed to examine changes to the ACA wall structure with age and disease progression.

## Materials and methods

### Subjects and sample preparation

A total of 59 ACA sections harvested from 50 human male brain donors were obtained from the Understanding Neurologic Injury and Traumatic Encephalopathy (UNITE) study at Boston University. Informed consent for all participants was obtained under protocols approved by the institutional review boards at Boston University and the VA Bedford Healthcare System (Bedford, MA). The methods for UNITE have been described previously (Mez et al., 2015). Briefly, UNITE is designed to characterize the long-term clinical and neuropathological effects of RHI, including but not limited, to CTE. It is composed of brain donors who are required to have a history of RHI from contact and collision sports, military service, physical violence or other sources. Symptomatic status is not part of the eligibility criteria. Brain donors are excluded if they had a prolonged *postmortem* interval or poor tissue quality. Brain donations are made by next of kin, referrals from medical examiners, the Concussion Legacy Foundation, or by the individuals prior to death. Comprehensive neuropathological evaluations are conducted, blinded to clinical data.

The brain tissue of each subject was semi-quantitively assessed for density of Aβ plaques and p-tau depositions using previously defined procedures and metrics for CTE (McKee et al., 2013) and AD (Hyman et al., 2012; Standring et al., 2019). CTE was diagnosed based on consensus criteria (McKee et al., 2016; Bieniek et al., 2021). Staging was based on regional p-tau involvement according to the McKee staging system since this has been shown to correlate with the duration of play and clinical symptoms (McKee et al., 2013; Alosco et al., 2020). For this study, stages were dichotomized into low (none, I and II) and high (III and IV) groups, which shows good agreement with low and high stages using consensus criteria (McKee et al., 2013; Alosco et al., 2020). Pathological AD was diagnosed separately, quantifying the spread of biomarkers including diffuse and neuritic Aβ plaque deposition as well as neurofibrillary tangle (NFT) formation (Hyman et al., 2012). Diffuse Aβ plaque progression was quantified using Thal phases as described in (Thal et al., 2002) and dichotomized into low (none, Thal phases 1-2) and high (Thal phases 3-5). Neuritic Aβ plaques were quantified via the CERAD criteria (Mirra et al., 2024) and dichotomized as low (no or sparse neuritic plaques) and high (moderate or frequent neuritic plaques). Spread of NFTs comprised of p-tau were staged as described in (Braak and Braak, 1991) and categorized as low (none to Braak stage II) and high (Braak stages III-VI). Overall AD progression was diagnosed using the NIA-Reagan criteria (Hyman and Trojanowski, 1997). The NIA-R, which considers the Braak stage and CERAD criteria, was determined and is presently dichotomized into low (none, low) and high (intermediate, high) probabilities of AD. Additionally, the spread of CAA (Standring et al., 2019) was dichotomized into low (score of 0–1) or high (score of two–3) groups. Distribution of the neuropathological measures associated with the ACA sections are included in Table 1. Prevalence of other neuropathologies were also assessed including LATE and Lewy body dementia (McKee et al., 2013), though not reported in the current study due to limited prevalence in the cohort.

**TABLE 1:** Subject age and neuropathology distributions including groups for each measure.

Measure	Group	Condition	n (%)
Thal Phase	Low	0	16 (32%)
		I-II	10 (20%)
	High	III	11 (22%)
		IV-V	13 (26%)
Braak Stage	Low	0	8 (16%)
		I-II	11 (22%)
	High	II-VI	19 (38%)
		V-VI	12 (24%)
CERAD Criteria	Low	None	27 (54%)
		Sparse	12 (24%)
	High	Moderate	7 (14%)
		Frequent	4 (8%)
NIA-Reagan Criteria Probability	Low	None	28 (56%)
		Low	11 (22%)
	High	Intermediate	7 (14%)
		High	4 (8%)
CAA Severity Score	Low	0	28 (56%)
		1	2 (4%)
	High	2	14 (28%)
		3	6 (12%)
CTE Stage	Low	0	9 (18%)
		I-II	6 (12%)
	High	III	19 (38%)
		IV	16 (32%)
Age	Young	≤65	18 (36%)
	Old	66-79	18 (34%)
	Oldest	≥80	15 (30%)

### Biaxial extension-inflation testing and data analysis

**FIGURE 1:**
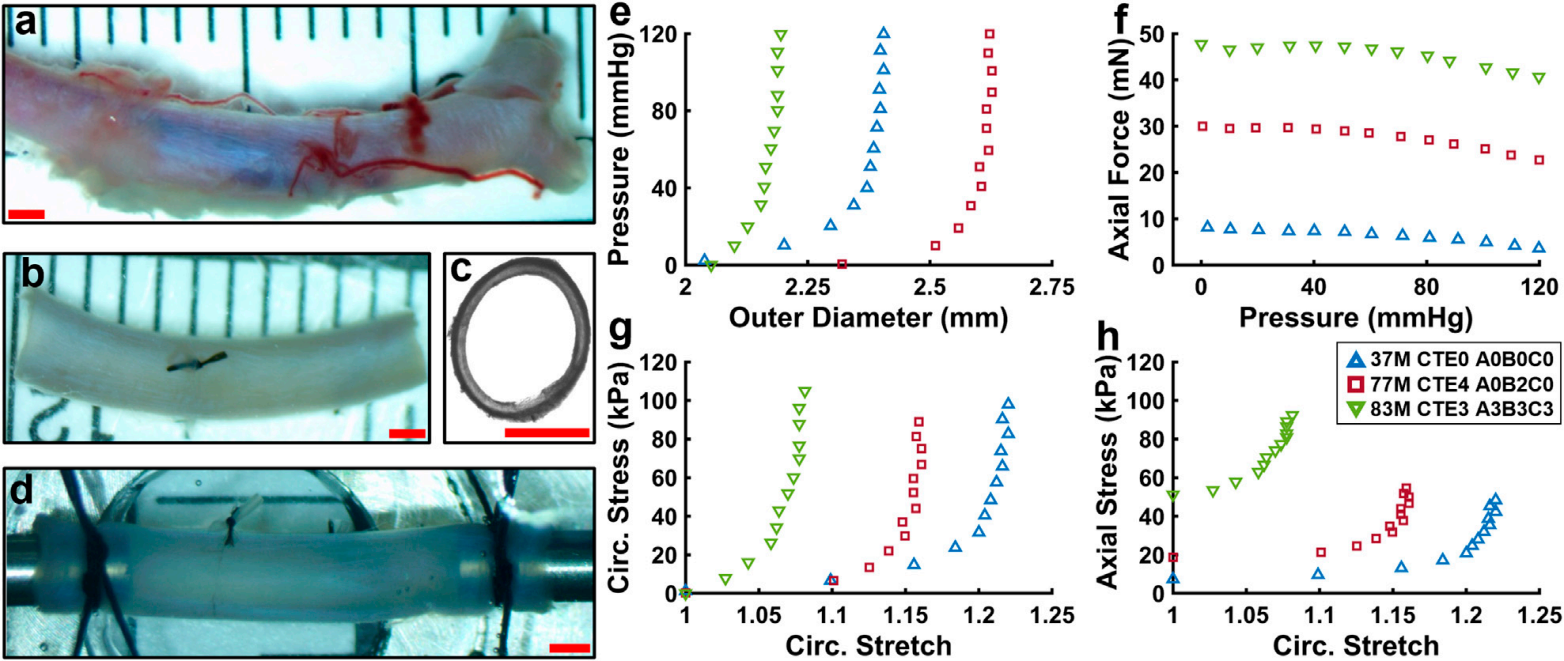
**(a–d)** Representative images of a human ACA during the experimental procedure. **(a,b)** ACA before and after the removal of connective tissue, side branches, and blood. **(c)** Thin ring cut from ACA to measure undeformed geometries. **(d)** ACA mounted on stainless steel cannula and axially stretched to its *in vivo* stretch ratio. **(e–h)** Representative biaxial inflation-extension data for three samples representing different ages and disease states. **(e)** Pressure-outer diameter response. **(f)** Axial force-pressure response. **(g,h)** Circumferential and axial stress responses.

ACA sections were isolated from fresh brains and stored in a −80 °C freezer prior to biomechanical testing and imaging. ACA samples 10–15 mm long were cleared of connective tissue and side branches were ligated using 7–0 nylon suture (Braintree Scientific Inc., Braintree, MA). Samples were then mounted on stainless steel cannula using nylon 6–0 suture (Fine Science Tools Inc., Foster City, CA) in a pressure myograph system (Danish Myo Technology A/S, Hinnerup, Denmark). Samples were pressurized using 1× phosphate-buffered saline (PBS) to characterize their passive mechanical properties under biaxial inflation-extension loading conditions. The myograph chamber was filled with PBS and heated to 37 °C. Representative images of the ACA during the experiment are shown in Figures 1a–d. The axial force $F_{z}$ , transmural pressure P , and deformed outer diameter $r_{o}$ were monitored during testing. Reference length of the ACA sample was measured between sutures and determined as the length at which $F_{z}$ was 0 mN. Cannulated sections were stretched to their *in vivo* axial stretch, defined as $\lambda_{z}=l/L$ , where *l is* the deformed (*in vivo*) length and *L* is the undeformed reference length. The *in vivo* axial stretch was estimated by minimizing force variation during the inflation-deflation cycle (Gkousioudi et al., 2024). Preconditioning constituted one inflation-deflation cycle to 100 mmHg and another to 120 mmHg. Four pressurization cycles to 120 mmHg were then performed to ensure reliability (Ferruzzi et al., 2013), and the final cycle was used for subsequent analysis.

Following mechanical testing, ring-shaped sections approximately 0.5 mm thick were cut from the samples and imaged to obtain the inner ( $R_{i}$ ) and outer ( $R_{o}$ ) reference radii. Assuming tissue incompressibility (Carew et al., 1968; Humphrey, 2002), deformed inner radius $r_{i}$ was obtained in Equation 1 as:

$$r_{i}=\sqrt{r_{o}^{2}-\frac{R_{o}^{2}-R_{i}^{2}}{\lambda_{z}}}$$ (1)

Circumferential stretch $\lambda_{\theta }$ was calculated in Equation 2 as:

$$\lambda_{\theta }=\frac{r_{o}+r_{i}}{R_{o}+R_{i}}$$ (2)

Assuming the artery to be a thin-walled cylinder, the Cauchy stresses in the axial and circumferential directions were determined using the following equations in Equation 3:

$$\sigma_{z}=\frac{F_{z}+P\pi r_{i}^{2}}{\pi \left(r_{o}^{2}-r_{i}^{2}\right)},\text{and }\sigma_{\theta }=\frac{Pr_{i}}{r_{o}-r_{i}}$$ (3)

Circumferential stretch $\lambda_{\theta }$ was normalized by dividing $\lambda_{\theta }$ at each pressure interval by the $\lambda_{\theta }$ at 0 mmHg, ensuring the stretch was one during pressurization. Initial modulus of the circumferential stress-stretch response was determined as the slope of a linear-fitted curve to the stress-stretch data between 0 and 20 mmHg.

Mechanical data were grouped by age (≤65, *n* = 18; 66–79, *n* = 17; and ≥80 years, *n* = 15) to examine age-related changes, then grouped by neuropathological scoring metrics to further examine correlations between ACA mechanical responses with pathological disease progression. These metrics were dichotomized into two groups for low and high levels, described in Table 1. Subjects under 65 years of age were removed when grouping by neuropathological measures to reduce advanced aging effects. Average stress-stretch curves were calculated for each group by averaging the stress and stretch measures at each pressure.

### Histological staining

The structure of the ACA wall was examined with histological staining. Briefly, a small segment of mechanically characterized ACA sample was cut and fixed with 4% paraformaldehyde in PBS overnight before being embedded in paraffin wax. Circumferential cross-sections 5 µm thick were cut and stained with Movat’s pentachrome to examine the cellular and extracellular components as well as the ground substance within the ACA wall.

### Multiphoton microscopy and image quantification

Multiphoton images were acquired using a FVMPE-RS confocal microscope (Olympus Life Sciences, Inc) equipped with a ×25 water-immersion objective lens (NA 1.05). The femtosecond IR pulse laser was set to 805 nm to generate two-photon excitation fluorescence (2PEF) signals of elastin (495–540 nm) and second harmonic generation (SHG) signals of collagen (390–420 nm) (Chow et al., 2014; Yu et al., 2018). Ring sections approximately 3 mm long were cut from the mechanically tested ACAs and submerged in PBS to examine the structure from the adventitial and intimal sides as well as the circumferential cross-section. SHG images of the adventitia were analyzed using the OrientationJ plugin within FIJI (Schindelin et al., 2012) to quantify the depth-dependence of collagen fiber orientation. Additionally, adventitial collagen fiber straightness was quantified using CT-FIRE (Bredfeldt et al., 2014) for samples from the ≤65 (*n* = 7), 66–79 (*n* = 4), and ≥80 (*n* = 6) age groups.

### Statistical analysis

Biaxial inflation-extension test data and biomechanical properties ( $F_{z}$ , P , $\sigma_{z,\max}$ , $\sigma_{\theta ,\max\;}$ , $\lambda_{\theta ,\max}$ , $\lambda_{z}$ , force variation), initial circumferential modulus, and geometric measures ( $R_{o}$ , $R_{i}$ , thickness) were tested for normality with the Shapiro-Wilk test. Data was statistically compared using one way ANOVA (age groups) or unpaired two-tailed *t*-tests (neuropathological groups). A *post hoc* analysis was performed with a Bonferroni correction to examine statistical significance when using ANOVA. General linear modelling was performed to assess the individual effects of each neuropathological measure (CTE, Thal, Braak, CERAD, CAA, NIA-R) on $\lambda_{\theta }$ at 120 mmHg, each model including age as a covariate. The effect of the independent variables (age, neuropathological measures, i.e., NPM) on $\lambda_{\theta }$ was quantified using the partial $\eta^{2}$ value from linear modelling, and their ratio $\eta_{NPM}^{2}/\eta_{age}^{2}$ was used to describe the effect of each measure relative to age. Data was considered significant when *p* < 0.05. Results are reported as mean ± standard error unless otherwise specified. Goodness of fit *R* ^2^ was calculated for linear regression analysis. All statistical analyses were performed using SPSS version 29.0.2 (IBM Corp, Armonk, NY).

## Results

Figures 1e–h show representative biaxial mechanical response for three human ACAs from subjects at different ages and neuropathological disease states. Outer diameter (Figure 1e) and axial force (Figure 1f) during pressurization vary between samples. Normalizing the biaxial inflation-extension data further reveals differences between ACA samples (Figures 1g,h). Most of the circumferential deformation occurs at sub-physiological pressures, with little change in circumferential stretch occurring above 60 mmHg (Figure 1g). Circumferential stretch appears to decrease in the older and diseased subjects implying a stiffening trend (Figure 1g).

**FIGURE 2:**
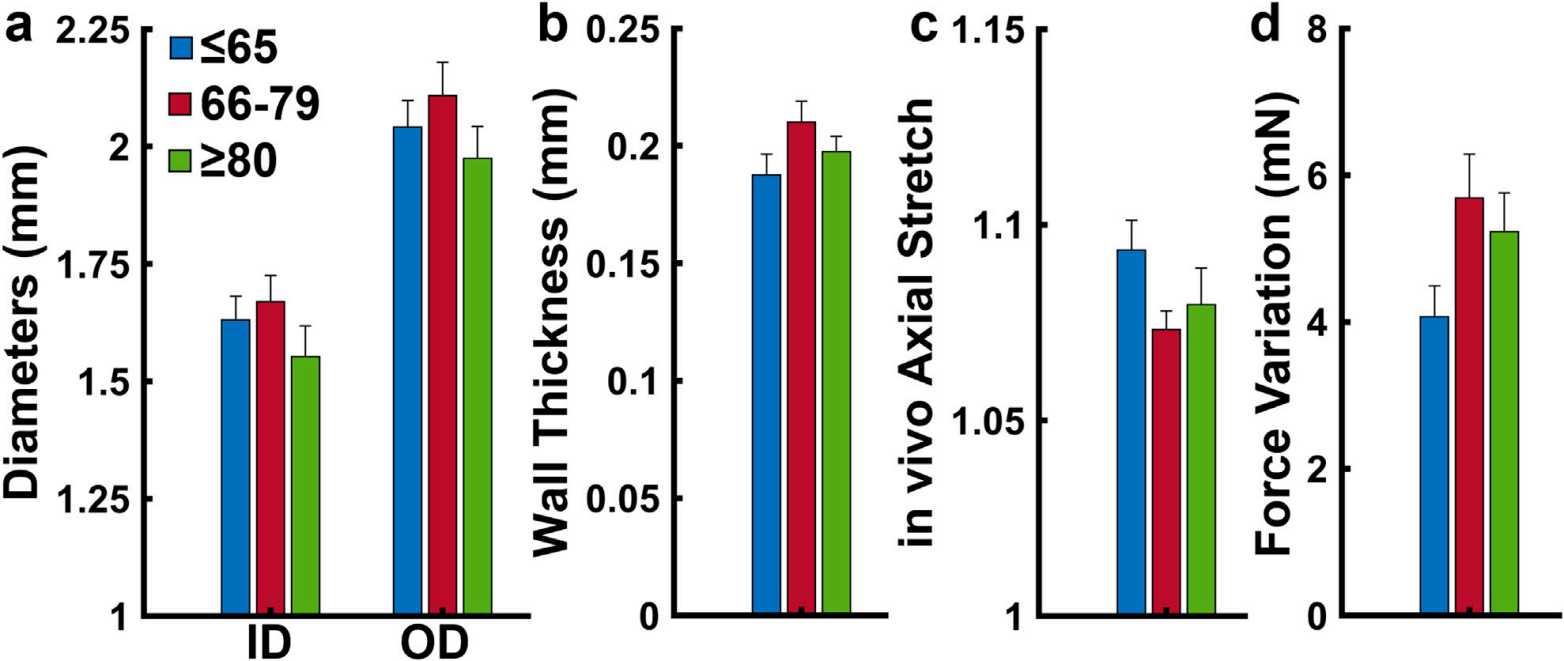
**(a)** Average inner and outer diameters **(b)** wall thickness, **(c)** *in vivo* axial stretch, and **(d)** variation in axial force during pressurization grouped by age (≤65, *n* = 18; 66–79, *n* = 17; ≥80 years, *n* = 15). Average values are presented as mean ± SEM. (*p < 0.05).

The undeformed outer diameters were 2.02 ± 0.05 mm, 2.10 ± 0.06 mm, and 2.00 ± 0.07 mm for the ≤65, 66–79, and ≥80 age groups, respectively (Figure 2a). The undeformed thickness increased from 204 ± 8 µm in the ≤65 years group to 219 ± 9 µm in the 66–79 age group and then decreases slightly to 213 ± 8 µm in the ≥80 group (Figure 2b). The *in vivo* axial stretch ratio was similar for all age groups, with values being 1.10 ± 0.01 for the ≤65 group, 1.07 ± 0.01 for the 66–79 group, and 1.08 ± 0.01 for the ≥80 group (Figure 2c). Variation in axial force during pressurization was 4.07 ± 1.79 mN, 5.69 ± 2.45 mN, and 5.23 ± 2.04 mN for the ≤65, 66–79, and ≥80 age groups, respectively (Figure 2d). None of the changes are significant.

**FIGURE 3:**
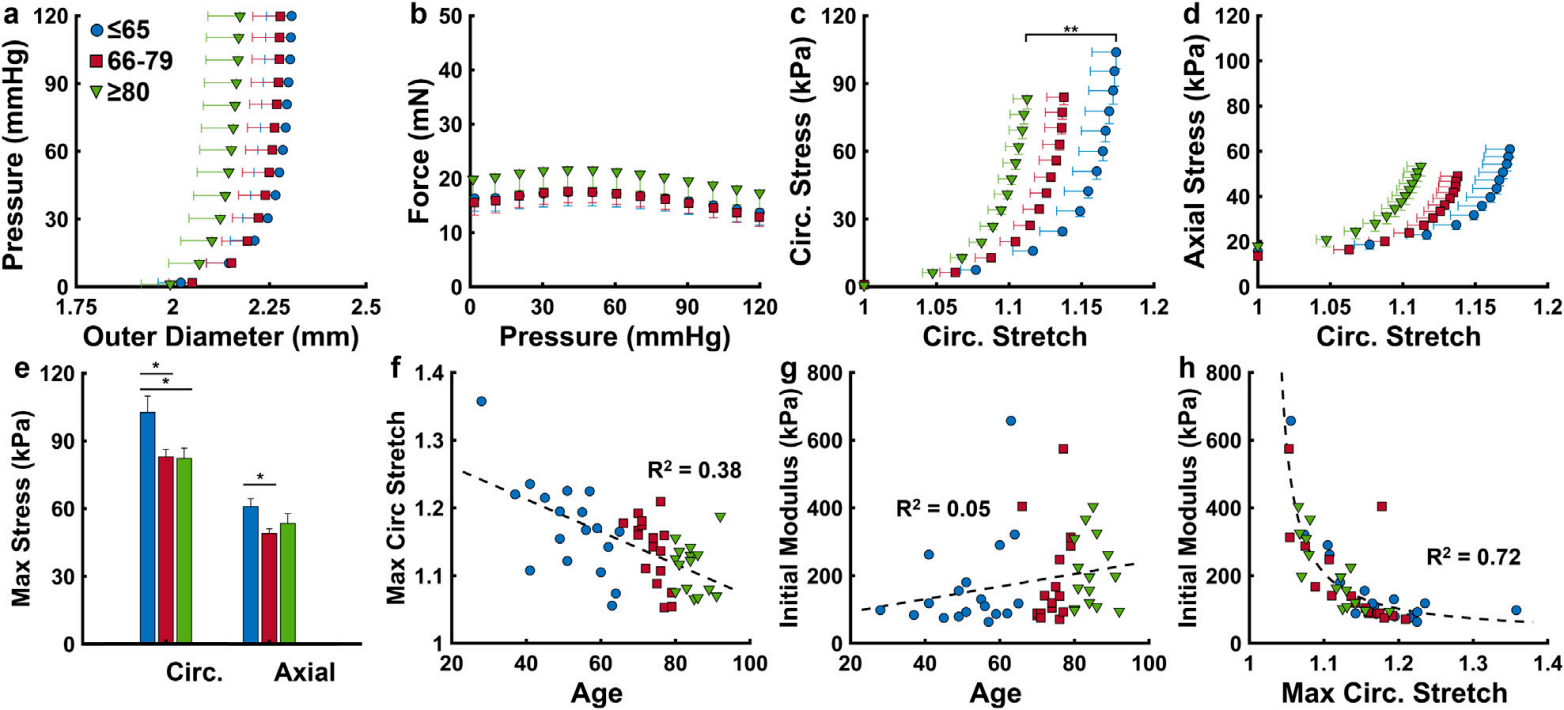
ACA mechanical response grouped by age (≤65, *n* = 18; 66–79, *n* = 17; ≥80 years, *n* = 15). **(a,b)** Average pressure-outer diameter and axial force-pressure response at the *in vivo* axial stretch ratio. **(c,d)** Average Cauchy stress-stretch behavior in the circumferential and axial directions. **(e)** Cauchy stress at 120 mmHg in the circumferential and axial directions. **(f,g)** Scatter plots describing effects of age on maximum circumferential stretch and initial modulus, calculated as the slope of a linear fit between 0 and 20 mmHg of the circumferential stress-stretch curve **(c)**. **(h)** Nonlinear relationship between the initial modulus and maximum circumferential stretch. Average values are presented as mean ± SEM. (*p < 0.05, **p < 0.01).

The outer diameter and axial force response during pressurization were plotted for the three age groups in Figures 3a,b. Notably, the oldest group experiences lower increases to the outer diameter during pressurization and has higher axial forces throughout. The Cauchy stress in the circumferential and axial directions plotted against the circumferential stretch reveal a progressive decrease in maximum circumferential stretch with aging (Figures 3c,d), including a significant difference between the ≤65 and the ≥80 age groups (*p* < 0.01). Circumferential stress is significantly higher in the ≤65 age group (103.9 ± 7.3 kPa) than both the ≤66–79 (83.9 ± 3.3 kPa) and ≥80 (83.2 ± 4.6 kPa) age groups (Figure 3e) (*p* < 0.05). The axial stress decreases significantly from the ≤65 age group (60.9 ± 3.6 kPa) to the 66–79 age group (49 ± 2.1 kPa) (*p* < 0.05), then increases slightly in the ≥80 group (53.4 ± 4.3 kPa) (Figure 3e). As maximum circumferential stretch (at 120 mmHg) decreases with age (Figure 3f), initial circumferential modulus (0–20 mmHg) slightly increases with age, from 167 ± 34 kPa to 182 ± 34 kPa to 220 ± 27 kPa for the ≤65, 66–79, and ≥80 age groups, respectively (Figure 3g). There exists a strong inverse correlation ( $R^{2}=$ 0.72) between the initial modulus and maximum circumferential stretch (Figure 3h).

**FIGURE 4:**
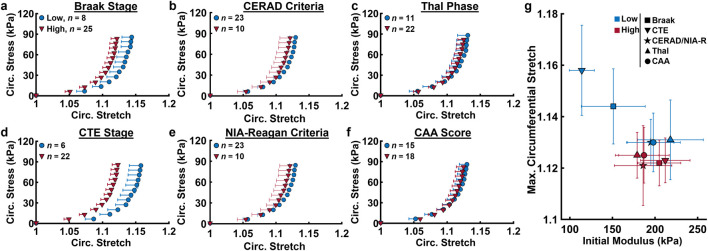
Circumferential Cauchy stress-stretch data from biaxial inflation-extension test at the low and high levels of various neuropathological measures. **(a)** Braak stage **(b)** CERAD stage, **(c)** Thal stage, **(d)** CTE stage, **(e)** NIA-Reagan Criteria probability, **(f)** cerebral amyloid angiopathy severity score. **(g)** Averaged maximum circumferential stretch vs. initial modulus derived from **(a–f)** for the low and high levels of each neuropathological measure. Average values are presented as mean ± SEM.

Averaged ACA mechanical response grouped by neuropathological biomarkers is shown in Figure 4, with samples below the age of 65 removed. Age distributions between groups for low and high severity of each measure was found to be statistically insignificant using t-tests. Elevated levels of measures of tau pathology (Braak, CTE stages) coincided with circumferential stiffening of ACAs, indicated by the leftward shift of the stress-stretch curve in brain donors with high levels compared to low levels for each measure (Figures 4a,d). Meanwhile, measures emphasizing Aβ plaques (Thal phase, CAA score, CERAD criteria) did not coincide with circumferential ACA stiffening, as indicated by the similarity in stress-stretch response when grouped by low and high amounts of these measures (Figures 4b,c,f). Additionally, grouping by low and high probabilities of AD based on the NIA-R criteria showed little correspondence with ACA circumferential response. The maximum circumferential stretch and initial modulus derived from Figures 4a–f were plotted in Figure 4g. Consistent with the results shown in Figures 4a–f, grouping by Braak and CTE stages reveals and increased initial modulus and decreased $\lambda_{\theta }$ in the ACAs as the disease progresses, while the other neuropathological measures show no statistically significant changes.

Table 2 contains a summary of the effects of each neuropathological measure on $\lambda_{\theta }$ for samples above the age of 65. The mean values for groups with low and high severities of each measure are presented along with a statistical parameter *p*, none of which are significant (*p* < 0.05). The covariation effect age is shown by the ratio $\eta_{NPM}^{2}/\eta_{age}^{2}$ , which describes the relative effect of each neuropathological measure relative to age. Like the mechanical data in Figure 4, the CTE and Braak stages have the largest effects on $\lambda_{\theta }$ relative to age (0.213 and 0.065 respectively), though both are still minor compared to age itself (5 times and 15 times lower, respectively). The Thal, CERAD/NIA-R, and CAA measures have much smaller effects relative to the CTE and Braak stages, having $\eta_{NPM}^{2}/\eta_{age}^{2}$ values of 0.021, 0.054, and 0.010, respectively, indicating that the effects of age are roughly 48, 31, and 100 times greater than these Aβ-driven measures.

**TABLE 2:** Summary of linear regressions between individual neuropathological progression effects on circumferential stretch λ_θ_ at 120 mmHg. Age accounted for in linear model as a covariate, and the relative effect of each measure compared to age is shown as a ratio of their $\eta^{2}$ values.

Neuropathological Measure	Group	Age (years)	λ_θ,120 mmHg_	*p*	*Relative effect size η* ^*2*^ */η*_*age*_^*2*^
CTE Stage	Low	72.7 ± 1.8	1.158 ± 0.018	0.056	0.213
	High	79.6 ± 1.3	1.120 ± 0.008		
Thal Phase	Low	78.2 ± 2.04	1.131 ± 0.015	0.648	0.021
	High	78.4 ± 1.5	1.125 ± 0.009		
Braak Stage	Low	75.1 ± 2.2	1.144 ± 0.015	0.203	0.065
	High	79.3 ± 1.4	1.126 ± 0.009		
CERAD/NIA-R Criteria	Low	78.2 ± 1.5	1.130 ± 0.009	0.592	0.032
	High	78.6 ± 2.2	1.121 ± 0.016		
CAA Score	Low	78.1 ± 1.8	1.1298 ± 0.011	0.744	0.01
	High	78.8 ± 1.6	1.1248 ± 0.011		

**FIGURE 5:**
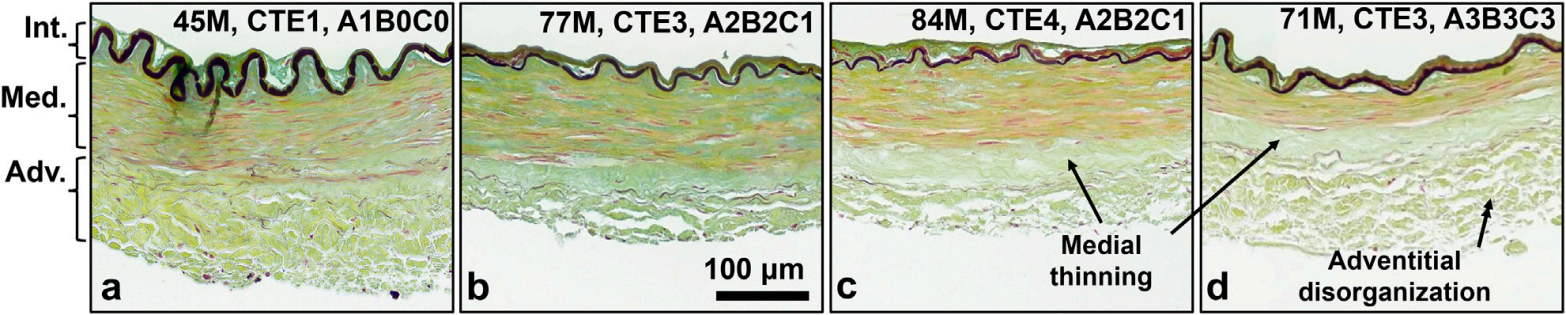
**(a-d)** Representative histology images of human ACAs at different ages and neuropathological disease stages. Elastin (dark grey) is primarily located in the internal elastic lamina, with sparse fibrous elastic content at the media-adventitia border. SMC nuclei (red), located in the medial section are elongated and oriented circumferentially. Collagen (yellow) is present in adventitia and media. Ground substance (blue) makes much of the wall appear green due to mixture with collagen. **(c,d)** Collagen disorganization in adventitia and SMC atrophy with medial thinning are both present in arteries from older and more diseased subjects compared to the younger and less diseased subjects in **(a,b)**.

Representative histological images depict the cross-sectional view of the ACA wall (Figure 5). Elastic fibers (grey/black) comprise the internal elastic lamina (IEL) and sparsely line the media-adventitia border. Smooth muscle cells (SMCs) occupy most of the medial layer, with their nuclei stained in red. Collagen (yellow) comprises much of the adventitia and media. Ground substance (blue) throughout the wall leads to a light green appearance overall due to mixing of the blue and yellow stains. In older subjects with further AD progression, the medial layer appears to decrease in thickness due to loss of SMCs and elastic fibers, leading to a band with only collagen at the media-adventitia border (Figures 5c,d). Adventitial collagen appears to be more disorganized and loosely packed (Figures 5c,d).

**FIGURE 6:**
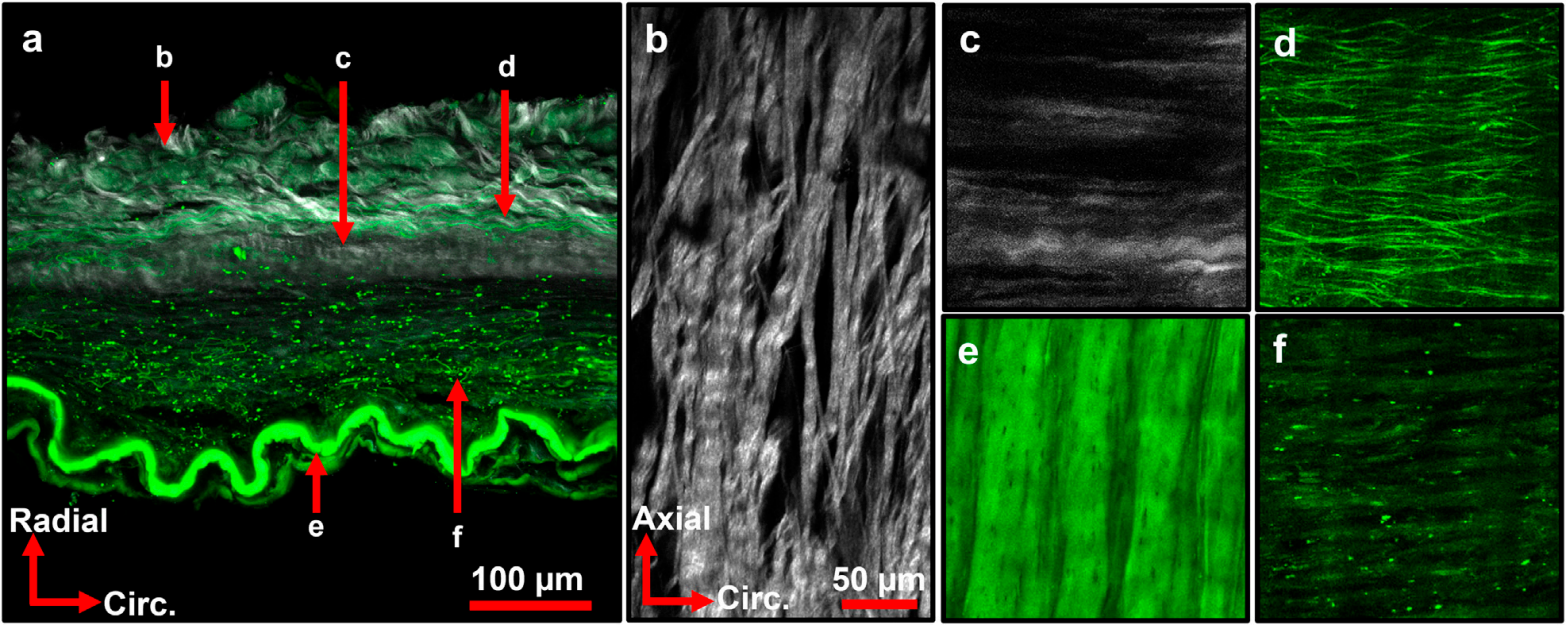
Representative multiphoton images of ACA wall depicting various elastin (green) and collagen (grey) structures. **(a)** Maximum intensity projection of ACA cross section. Arrows indicate local region imaged in **(b–f)**. **(b)** Adventitial collagen primarily aligned in axial direction with crimping present. **(c)** Collagen bundles at media-adventitia border primarily aligned in circumferential direction. **(d)** Elastic fibers at media-adventitia border primarily aligned in circumferential direction. **(e)** Internal elastic lamina with crimped structure and small fenestrations present. **(f)** Elastin signal from the media, with sparse elastic content. **(c–f)** Share orientation axes and scale bar with **(b)**. Image **(a)** is from a 92M subject with CTE3, A2B2C2. Images **(b,e,f)** are from an 84M subject with CTE4, A3B3-C2. Images **(c,d)** are from a separate 84M subject with CTE4, A3B2C1.

**FIGURE 7:**
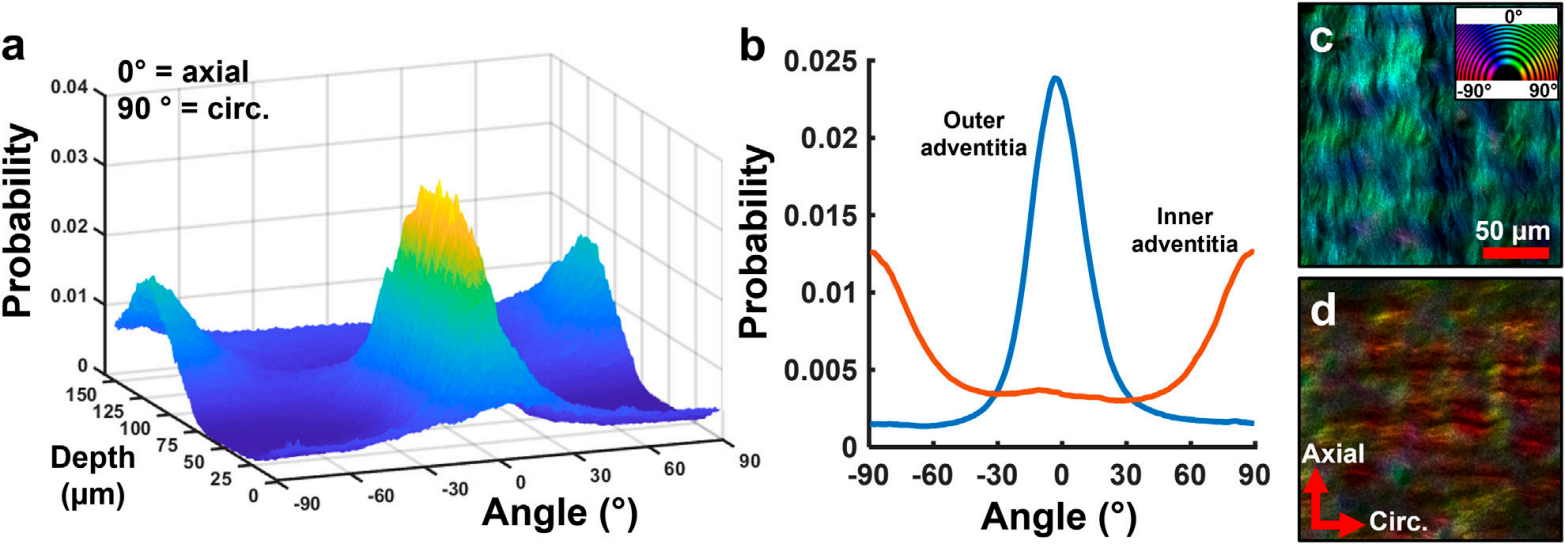
Representative study on depth-dependence of collagen orientation in human ACA. **(a)** 3D surface plot of collagen orientation distribution through the thickness shows a transition from circumferential to axial orientation from the inner to the outer adventitia. **(b)** Average orientation distribution of collagen in the inner and outer adventitia from **(a)**. **(c,d)** Representative single slice images of the **(c)** outer and **(d)** inner adventitia showing distinct collagen orientations. Color wheel indicates fiber orientation.

Representative multiphoton microscopy images further revealed the elastic and collagen fiber network structures in the ACA (Figure 6). A circumferential-radial cross section (Figure 6a) like those in Figure 5 shows the distribution of elastin and collagen throughout the thickness of the wall. Apart from the IEL at the intima (Figures 6a,e), fibrous elastin content in cerebral arteries is mostly found at the adventitia-media border (Figure 6d) with scattered elastin in the media (Figure 6f). The IEL is a thin, corrugated sheet of densely woven elastic fibers with small fenestrations present (Figure 6e). The circumferential-longitudinal orientation of the medial elastin (Figure 6f) in conjunction with the circumferential-radial cross-section (Figure 6a) confirms that the stronger elastic signal in the media appears scattered. The adventitial collagen bundles in the outer media are primarily aligned in the axial direction (Figure 6b) with some dispersion present. However, collagen bundles at the media-adventitia border are mostly circumferentially oriented (Figures 6c,d). The transition from axial to circumferentially oriented collagen fibers in the adventitia was seemingly abrupt, as shown in the fiber orientation distribution function in Figure 7.

**FIGURE 8:**
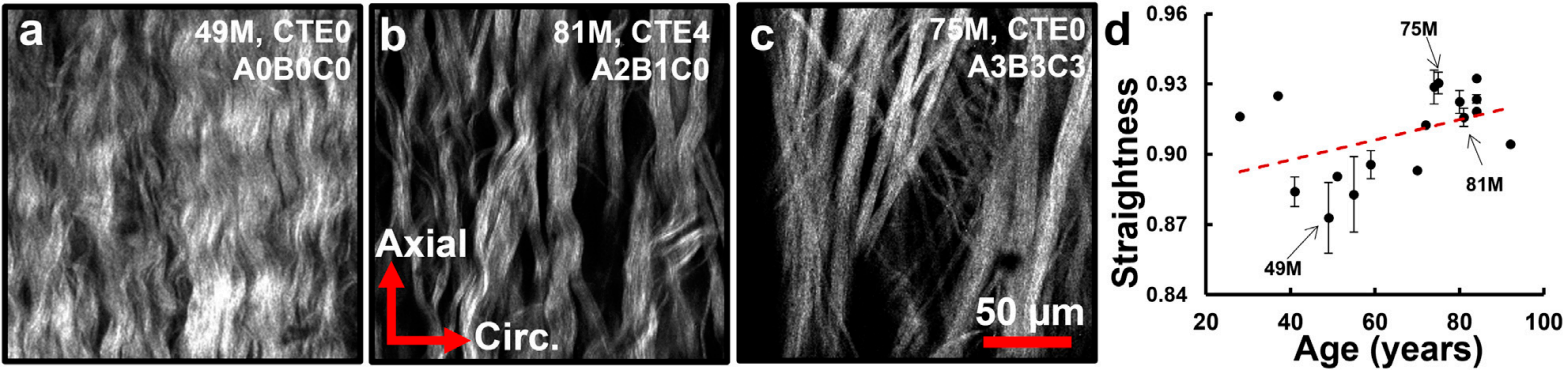
**(a–c)** Representative single scan images of adventitial collagen bundles with varied fiber straightness from subjects at different ages and neuropathological disease stages. **(d)** Computed median fiber straightness parameter (*n* = 18) displays a trend of fiber straightening with age (*R* ^2^ = 0.196).

Multiphoton images of collagen bundles at the outer adventitia for multiple ACA samples were acquired with three representative subjects of different ages and disease states shown in Figures 8a–c. Collagen fibers appear more crimped in the younger samples (Figure 8a) and straighter in the older, more diseased samples (Figure 8c). Straightness parameter analysis reveals fiber straightening with age (Figure 8d). Median fiber straightness increases from 0.897 in the ≤65 age group to 0.917 and 0.920 in the 66–79 and ≥80 age groups, respectively, with a significant difference reported between the ≤65 and ≥80 age groups (*p* = 0.01).

## Discussion

Growing evidence suggests vascular disease underlies AD onset and progression. Using coupled biomechanical and microstructural analyses, our study provides insightful findings on the relationship between compromised mechanical function and remodeling of the ACA. Large cerebral arteries play a crucial role in dampening the pulsatile pressure to protect small vessels downstream and the BBB but are subject to structural changes including SMC atrophy, elastin degradation and collagen reorganization with age and neuropathological disease. The present findings of ACA structural and functional changes shed light on the complex interactions between cerebrovascular remodeling, aging, and progression of neurodegenerative diseases.

Cardiovascular aortic stiffening with age is well documented and is closely associated with the progression of many diseases (McEniery et al., 2007; Kohn et al., 2015). However, much is still unknown about cerebrovascular remodeling in aging and its role in neurodegenerative disease onset. Our study shows that human ACAs become progressively stiffer with age (Figures 3c,f,h), and that this change is manifested by an increase in the initial tangent modulus during pressurization leading to a decrease in maximum circumferential stretch. Previous studies have reported age-related stiffening of various cerebral arteries using *in vitro* mechanical testing techniques (Busby and Burton, 1965; Hayashi et al., 1980). More recently, Monson et al. examined axial failure properties and biaxial mechanics of human cerebral vessels, finding no significant effects of aging on the mechanical response (Monson et al., 2005; 2008), though this discrepancy could be due to variability in human tissue accompanied by smaller sample sizes, or differences in remodeling of the axial and circumferential directions.

Examining the ACA microstructure further helps us understand the changes in their mechanical function. Insignificant changes to undeformed diameters, wall thickness, and axial stretch with age (Figures 2a–c) despite ACA stiffening (Figures 3c,f) suggest remodeling in the ACA wall. Histological staining revealed SMC atrophy and medial thinning in ACAs from neuropathologically-affected aged subjects (Figures 5c,d). SMC atrophy in large cerebral arteries has been well documented (Perry et al., 1998; Fonck et al., 2009; Liu et al., 2023). In (Perry et al., 1998), the researchers reported a significant reduction in medial SMC layer thickness in AD patients compared to age-matched controls, with no correlation between CAA and SMC thinning, suggesting that SMC atrophy may occur through Aβ-independent mechanisms. Fonck et al. (2009) similarly observed reduced SMC content accompanied by increased medial collagen deposition, indicative of structural remodeling. In our previous work (Liu et al., 2023), we identified pronounced SMC atrophy concentrated at the media-adventitia interface, resulting in overall medial thinning, consistent with the current data (Figures 5c,d). The cohort in our previous study (Liu et al., 2023) also employed age-matched subjects without a history of RHI, further supporting the location-specific SMC loss and medial thinning are associated with AD-related vascular degeneration rather than aging or trauma-related factors. Collagen crimping is vital to wall compliance which allows the vessel to expand and accommodate pulsatile blood pressure and brain perfusion (Holzapfel et al., 2000). Straightening of adventitial collagen with age, evidenced by SHG images and quantified straightness parameters (Figure 8), likely contributes to the earlier stiffening of the ACA wall during pressurization (Figures 3g,h).

We further examined the relationship between AD- and CTE-associated biomarker progression and cerebrovascular dysfunction. Our study found a relationship between ACA stiffening and increased formation of NFTs in CTE and AD (Figures 4a,d). Despite this, age is still the primary marker for ACA stiffening, as its effects are roughly 5 times greater than the CTE stage and 15 times greater than the Braak stage (Table 2.) Measures quantifying the levels of Aβ plaque formation (Thal phase, CERAD and NIA-R criteria, CAA score) had little correlation with ACA stiffening (Figures 4b,c,e–f) and showed negligible effects on $\lambda_{\theta }$ relative to age (Table 2).

The correlations between ACA stiffening with measures associated with NFTs but not Aβ plaques suggests that buildup of p-tau may be more closely associated with remodeling of ACA wall mechanics than Aβ. While Aβ plaques are known to form in small and medium-sized arteries which impact cerebral blood flow and perfusion (Standring et al., 2019), to our knowledge, they have not been observed in large cerebral arteries like the ACA. Emerging evidence implicates tau pathology, rather than Aβ deposition, as the potential driver of AD progression (Kametani and Hasegawa, 2018). Several studies reveal a strong correlation between NFT density and cognitive decline severity (Serrano-Pozo et al., 2011; Forner et al., 2017). In early-stage AD, synaptic dysfunction due to NFT formation is a key contributor to cognitive decline (Wu et al., 2021). Additionally, overexpression of tau was shown to induce cerebrovascular remodeling and perturbs cerebral hemodynamics (Bennett et al., 2018), establishing a mechanistic link between tauopathy and vascular changes. Meanwhile, formation of extracellular Aβ plaques is thought to be related to other age-related processes like buildup of oxidative stress (Cai et al., 2011; Huang et al., 2016).

Smooth muscle cell atrophy (Figure 5) and collagen straightening (Figure 8) observed presently may also be linked to AD- and CTE-related NFTs. Vascular SMCs play a critical role in modulating vascular tone through active contraction and relaxation, thereby accommodating dynamic changes in cerebral blood flow. These contractile dynamics also contribute to perivascular clearance of neurotoxic metabolites, including Aβ and tau. Age and AD associated reductions in SMC content (Figures 5c,d) can impact both hemodynamic regulation and waste clearance (Cockerill et al., 2018). Perivascular nerves, embedded extrinsically into the adventitia, exert critical neuromodulatory control over vascular contractility (Bleys and Cowen, 2001; Hamel, 2006). Degradation of these perivascular innervations in large cerebral arteries has been documented during AD progression (Bleys et al., 1996), and, when coupled with SMC loss, likely contribute to diminished vascular contractility and impaired clearance neurotoxic metabolites. Additionally, arterial stiffening has been shown to compromise perivascular clearance efficiency (Iadecola and Gottesman, 2018; Govindpani et al., 2019). In our data, increased collagen fiber straightness (Figure 8) elevates artery stiffness at lower pressures (Figure 3g), thereby reducing compliance and further limiting pulsatility-driven waste clearance.

It is important to note the potential impacts of RHI on the results of this study. All subjects in the current study are from the UNITE cohort and varying amounts of RHI exposure. While RHI is primarily connected to the development of CTE (McKee et al., 2013), many subjects were also assessed with multiple neuropathologies including AD. RHI exposure likely has impacts on brain perfusion and function. Apart from CTE, RHI is a known risk factor for several dementias including AD (Plassman et al., 2000). The physical trauma from head injuries likely triggers molecular pathways resulting in the unwanted accumulation of neurotoxic proteins like tau and Aβ (McKee et al., 2013). Pathological assessments of brain tissue have also correlated RHI with the occurrence of small cerebrovascular diseases independent of CTE tauopathies (Emrani et al., 2025). Noninvasive measurements in subjects with RHI exposure have reported region-specific alterations in blood flow both in mouse models (Ojo et al., 2016) and retired NFL players not showing clinical CTE symptoms (Hart et al., 2013). These findings suggest that RHI may independently impact cerebrovascular function, and further studies are needed to understand its causal role in the development of CTE and other neuropathologies.

The relationship between arterial biomechanics, aging, and neuropathological progression is complex. Separating the cognitive effects of neuropathologies from those of aging remains a significant challenge. Age is the primary risk-factor for dementias such as AD (Harman, 2006) and all pathologies require time to develop. Moreover, most dementia cases in older adults present with multiple coexisting neuropathologies (Schneider et al., 2007). Previous studies have documented frequent overlap among AD, CTE, cerebrovascular disease, and other dementia-related pathologies (Jellinger and Attems, 2007; McAleese et al., 2021; Saltiel et al., 2024; Emrani et al., 2025). These investigations have sought to correlate individual and mixed pathologies with clinical symptoms. However, the mechanistic pathways underlying the onset of most of these neuropathologies remain poorly defined. Large-scale biochemical, neurocognitive, and tissue compositional studies are needed to resolve the relative contributions of single and coexisting pathologies, and to clarify how vascular changes contribute to, or result from, these disease processes. In particular, comparative analyses of arteries from aged control and AD subjects without RHI or CTE will be essential to delineate the interplay among aging, neuropathologies, and cerebrovascular remodeling.

Note that our study does not allow conclusions about whether cerebrovascular remodeling precedes or results from neurodegeneration. Nonetheless, the observed changes in cerebrovascular mechanics and structure provide valuable context for previously reported noninvasive measurements of cerebrovascular dysfunctions. Multiple studies have noninvasively investigated age-related (Stoquart-ElSankari et al., 2007; Zhu et al., 2013; Wu et al., 2016; Jefferson et al., 2018) and disease-related (Roher et al., 2012; Hart et al., 2013) cerebrovascular dysfunctions, reporting altered cerebral blood flow, reduced cerebral blood volume, and impacted perfusion. The current study provides a mechanistic explanation for these observations–increased vessel stiffness (Figures 3, 4) reduces *in vivo* lumen area, which in turn decrease cerebral blood volume and disrupts flow dynamics. Notably, cerebral vessel stiffness has been shown to correlate with cognitive decline and may serve as a biomarker for cerebral small vessel disease (Singer et al., 2014), frequently observed in older and demented individuals (Chojdak-Łukasiewicz et al., 2021). Despite progress, noninvasive measurements of arterial stiffness, particularly in deep brain vessels, remain a challenge. Emerging elastography techniques (Li et al., 2017; 2022) may offer promising potential for future *in vivo* applications.

## Limitations

Acquisition of human cerebrovascular tissue remains a challenge and as such one must exercise caution by interpreting the results of this study. Performing a statistical power analysis for sample size, we found the comparisons for mechanical data between the three age groups to be slightly underpowered (0.74) relative to a standard sufficient power of 0.8. Power values for the Braak and CTE group sizes were also underpowered at 0.17 and 0.51, respectively, indicating that further studies are needed to better understand the relationship between cerebrovascular mechanics and these tauopathies. Additionally, the UNITE cohort primarily comprising male subjects with RHI exposure limits the generalizability of the findings in the study. Sex differences in vascular stiffening trends are well documented (DuPont et al., 2019; Kehmeier and Walker, 2021), and women account for nearly two-thirds of all AD patients in the United States (Rajan et al., 2021). Additionally, the physical trauma from RHI exposure in this cohort may be a confounding factor on cerebrovascular mechanics independent of the tauopathies characterizing CTE. Future studies are also required to further delineate the vascular and clinical effects of age and neuropathologies.

ACAs with minimal atherosclerosis were chosen for this study to eliminate the effects of atherosclerotic plaques on our mechanical characterizations. However, atherosclerosis in cerebral arteries is a known risk factor of AD and a significant increase of stenosis was reported in multiple cerebral arteries from AD-afflicted patients (Roher et al., 2003). These features impact brain hemodynamics and contribute to hypoperfusion, both of which can lead to mechanical and microstructural changes in the cerebral arteries (Monson et al., 2008). Structural quantification of the ACA remains a challenge. The elastic fibers at the adventitia-media border are generally ∼80–100 µm from the surface, beyond the penetration depth of multiphoton microscopy (50–60 µm). Further studies may use optical clearing techniques (Schriefl et al., 2013) to increase multiphoton microscopy penetration depth and to further investigate structural changes in the circumferentially oriented collagen and elastin closer to the media. Future biomechanical studies on fresh tissue are needed to more accurately understand the *in vivo* biomechanical response of cerebral arteries. Future investigations are also needed to better understand the structural and functional changes of other cerebral vessels, such as the middle cerebral artery, which may be relevant to the primary medial temporal lobe pathology in AD due to its proximity.

## Conclusion

With an aging population and increasing cases of AD and other neuropathological diseases, there is a need to understand cerebrovasculature remodeling due to its close relationship with the brain. This study provides new understandings on the mechanical response and microstructure of human ACAs, as well as the complex relationships between the cerebrovasculature and brain tissue with age and neurodegenerative disease. Our findings showed progressive age-related stiffening of human ACAs. Furthermore, accounting for the effects of age, ACA stiffening was evident from subjects with elevated levels of tau-based neuropathologies including neurofibrillary tangles, characteristic of both AD and CTE progression, though the effects of age were still prevalent. Microstructural studies of the ACA wall revealed decreased SMC content and straightening of adventitial collagen bundles with age and neuropathological changes, which likely contributed to the stiffened mechanical response. Understanding the role of cerebrovascular remodeling in neurodegenerative diseases may lead to the discovery of new treatment options and directions for interventions.

## Data Availability

The original contributions presented in the study are included in the article/supplementary material, further inquiries can be directed to the corresponding author.
